# Early seizures and heterogeneity of physiologic recovery in heat-related illness: a nationwide registry study

**DOI:** 10.1186/s13054-026-05961-7

**Published:** 2026-03-20

**Authors:** Koichi Inukai, Koki Terakawa, Takuya Matsui, Takayuki Hattori, Miyuu Uchida, Shiho Tane, Yu Hashimoto, Masanori Kawamoto, Fumitaka Kato, Koji Amano, Hiroyuki Kayata, Hideaki Yakushiji, Nobutaka Mukai, Naoki Shinyama, Akihiro Usui, Masanori Morita

**Affiliations:** Department of Trauma and Critical Care Medicine, Sakai City Medical Center, 1-1-1 Ebaraji-cho, Nishi-ku, Sakai, 593-8304 Osaka Japan

**Keywords:** Heat-related illness, Heatstroke, Early seizures, In-hospital mortality, Propensity score matching, Recovery dynamics, Nationwide registry

## Abstract

**Background:**

Generalized seizures in heatstroke have traditionally been interpreted as a clinical sign of severe central nervous system involvement. However, it remains uncertain whether seizures documented early in the disease course uniformly reflect irreversible injury or may be observed in patients with preserved short-interval physiologic responsiveness. We aimed to characterize early physiologic trajectories associated with early seizures and to explore their association with in-hospital mortality.

**Methods:**

We conducted a retrospective nationwide cohort study of adults hospitalized with heat-related illness in the Japanese Association for Acute Medicine Heatstroke and Hypothermia Surveillance (JAAM-HsS) registry from 2017 to 2024. Early seizures were defined as generalized seizures observed or documented during prehospital transport or the initial emergency department assessment. The primary objective was to compare Day 1–Day 2 short-interval trajectories of key metabolic, electrolyte, and fluid-balance markers by early seizure status using linear mixed-effects models, focusing on seizure-by-time interactions. In-hospital mortality was evaluated as a secondary observational outcome using prespecified multivariable logistic regression (with vs. without lactate) and 1:1 propensity score matching, with attention to negative confounding and survivorship-related bias.

**Results:**

Of 3,859 patients, 408 (10.6%) had early seizures. Patients with early seizures exhibited greater initial derangement (higher body temperature and lactate and lower base excess) yet showed more rapid short-interval normalization of base excess, lactate, glucose, and serum sodium by Day 2 (all interaction *p* < 0.05). In-hospital mortality was 180/3,451 (5.2%) without early seizures and 27/408 (6.6%) with early seizures. In multivariable models adjusting for measured covariates, early seizures were inversely associated with mortality (lactate-included: aOR 0.48, 95% CI 0.27–0.86; lactate-excluded: aOR 0.57, 95% CI 0.33–0.99). This inverse association was directionally consistent after propensity score matching.

**Conclusions:**

Early seizures in heat-related illness were associated with a distinct short-interval trajectory phenotype that may help calibrate early prognostication. An inverse association with in-hospital mortality was observed in adjusted and covariate-balanced analyses; however, this should be interpreted as a secondary marker–outcome association and may reflect residual or negative confounding, including differential seizure ascertainment among the most moribund patients. These findings are hypothesis-generating and warrant prospective validation with higher-granularity neurologic and treatment data.

**Supplementary Information:**

The online version contains supplementary material available at 10.1186/s13054-026-05961-7.

## Background

Heat-related illness is a life-threatening emergency that can culminate in multiorgan failure and death due to failure of thermoregulation [1]. Its global burden is increasing in parallel with climate change and population aging [2–5]. In severe heatstroke, central nervous system dysfunction is common, and impaired consciousness and generalized convulsive seizures have long been regarded as markers of disease severity [1, 6, 7].

Across diverse clinical contexts—including acute ischemic and hemorrhagic stroke, post–cardiac arrest syndrome, and bacterial and viral encephalitis—seizures have been reported as predictors of poor outcomes, including mortality and long-term neurological impairment [8–16]. In contrast, within the heatstroke literature, seizures have often been described as accompanying manifestations, and systematic investigations that substantiate the clinical interpretation of seizures in heat-related illness remain limited [1, 17–19]. Moreover, prior studies have seldom addressed the interplay between early systemic stress—such as metabolic, electrolyte, and circulatory derangements—and neurological events. Consequently, it remains unclear whether seizures uniformly signify irreversible central nervous system injury or may, in some patients, be observed as a surrogate marker of preserved neurologic reactivity under extreme hyperthermia.

In routine clinical practice, early generalized convulsions documented during prehospital transport and/or upon emergency department arrival are sometimes observed across a broad spectrum of illness severity and are not confined to the most moribund patients. This observation suggests that early seizures may be encountered not merely as a single severity marker, but as part of heterogeneous early physiologic response patterns—potentially reflecting distinct phenotypes present during the initial phase of hospitalization.

To address these knowledge gaps, we conducted descriptive and exploratory analyses using a nationwide multicenter registry. Specifically, we compared short-interval early physiologic recovery patterns by early seizure status, operationalized as trajectories of markers reflecting metabolic, electrolyte, and fluid dynamics during the first two hospital days. The association with in-hospital mortality was examined as a secondary observational outcome, with careful attention to the potential for negative confounding and survivorship bias, including under-ascertainment of seizures among the most critically ill. This study is hypothesis-generating and is not intended to support interpretation of early seizures as an outcome-improving factor. Rather, we sought to explore whether early seizures may function as a clinical marker of acute systemic state by delineating how they correlate with short-term early recovery patterns, thereby highlighting observable phenotypes that warrant prospective validation with higher-granularity neurologic and treatment data.

## Methods

### Study design and setting

This retrospective nationwide cohort study used the Japanese Association for Acute Medicine Heatstroke and Hypothermia Surveillance (JAAM-HsS), a multicenter registry of patients hospitalized with heat-related illness in Japan [20]. The JAAM Heatstroke and Hypothermia Surveillance Committee prospectively collects clinical information annually during the summer season (July through September) for patients admitted with heat-related illness.

The study was designed primarily to describe and compare early short-interval physiologic trajectories associated with clinically documented early generalized convulsions; mortality was evaluated as a secondary, observational outcome.

The study protocol was approved by the Teikyo University Ethics Committee for Medical and Health Research (approval number 17-021-5; “Heatstroke STUDY”; approval date May 21, 2020), with additional approvals obtained at participating institutions as required. The study was conducted in accordance with the Declaration of Helsinki and subsequent amendments. Data were anonymized in the registry; given the observational nature of the study, the requirement for informed consent was waived.

### Data source

At participating hospitals, physicians or trained staff extracted data from electronic medical records and prehospital transport documentation and entered information into standardized case report forms. Data were submitted via a web-based data capture system to the JAAM-HsS office, aggregated on a central server, and curated into an analytic dataset for research.

### Participants

We included adult patients (≥ 18 years) hospitalized with heat-related illness between July 1 and September 30 in each year from 2017 to 2024. Heat-related illness was defined as a spectrum from heat exhaustion to heatstroke associated with exertional or environmental heat stress. Diagnoses were made by local clinicians based on clinical manifestations and heat exposure history, in alignment with JAAM heatstroke guidance and the modified JAAM heatstroke criteria (mJAAM) [21, 22], and treatments were provided according to local clinical practice.

**Fig. 1:**
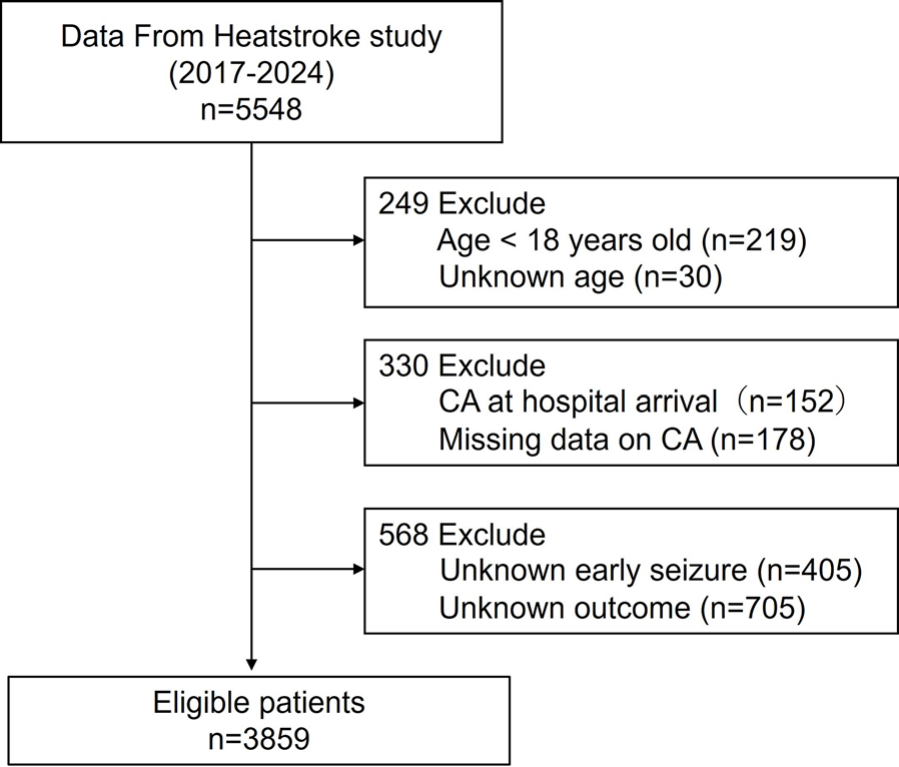
Flow diagram of patient selection. Flowchart showing patient inclusion and exclusion from the nationwide heatstroke registry

We excluded patients who (1) were in cardiopulmonary arrest on arrival, (2) had unknown in-hospital mortality status, or (3) lacked information on early seizures. Patients in cardiopulmonary arrest were excluded because admission vital signs and laboratory data were not considered reliably interpretable. The patient selection process and derivation of the final analytic cohort are shown in Fig. 1.

### Definitions

The study exposure was early seizures, defined as clinically documented generalized convulsive seizures recorded during prehospital transport and/or at emergency department arrival or during the initial emergency department assessment (i.e., up to completion of the initial emergency department evaluation). Localized muscle cramps (heat cramps) were not considered seizures.

Seizure classification was based on routine clinical documentation and did not include electroencephalographic confirmation or detailed semiology.

Patients were classified as having early seizures when generalized tonic–clonic activity was recorded by emergency medical services personnel or emergency department clinicians; all others were classified as no early seizures.

In this study, we use the term “reactivity” as a descriptive construct consistent with the registry design: an acute stress-response capacity and early short-term reversibility, empirically characterized by (i) the magnitude of early perturbation at initial clinical assessment (including salient clinical events such as early generalized convulsions and key physiologic markers reflecting metabolic and homeostatic domains) and (ii) the subsequent short-interval change in these markers between available time points. Given the registry’s limited temporal resolution, this “short-interval reversibility” is treated as an observed trajectory between discrete measurements rather than as mechanistic inference.

### Outcomes

The primary outcome was early short-interval physiological recovery patterns through hospital Day 2, evaluated as Day 1–Day 2 trajectories in metabolic, electrolyte, and fluid-related markers.

In-hospital mortality (death from any cause during hospitalization) was evaluated as a secondary outcome and analyzed as a binary endpoint; ICU and total hospital length of stay were also assessed as secondary outcomes.

### Variables

We extracted age, sex, height, weight, body mass index (BMI), onset location (outdoor/indoor), time from symptom onset to hospital arrival, prehospital and on-arrival vital signs (systolic and diastolic blood pressure, heart rate, respiratory rate, body temperature), level of consciousness assessed by the Glasgow Coma Scale (GCS), ICU and hospital length of stay, and in-hospital mortality.

We also extracted available comorbidity indicators recorded in JAAM-HsS (cardiovascular disease, respiratory disease, diabetes mellitus, and psychiatric disease) for use in a sensitivity propensity score matching analysis.

Body temperature was recorded as core temperature when available (rectal, bladder, or esophageal). When core temperature was unavailable, surface temperature (axillary or tympanic) was used. If the symptom onset time was unknown, the discovery time was used.

Variables labeled as “Day 1” represent the initial measurements recorded at emergency department arrival.

Systolic blood pressure corresponds to the initial value measured at emergency department arrival.

Admission (Day 1) laboratory measurements included pH, lactate, base excess (BE), electrolytes (sodium, potassium, chloride), white blood cell (WBC) count, platelet count, hemoglobin, hematocrit, creatinine, blood urea nitrogen (BUN), total bilirubin, aspartate aminotransferase (AST), alanine aminotransferase (ALT), creatine kinase (CK), C-reactive protein (CRP), and prothrombin time (PT) activity.

Variables labeled as “Day 2” correspond to a single measurement obtained on the following calendar day, typically representing routine follow-up testing.

### Statistical analysis

To characterize early short-interval recovery patterns associated with clinically documented early seizures (primary objective) and to explore the association with in-hospital mortality as a secondary observational outcome, we performed analyses in the following sequence: (1) descriptive statistics, (2) exploratory Day 1–Day 2 recovery-dynamics analyses, (3) unadjusted and prespecified multivariable logistic regression for in-hospital mortality, (4) propensity score matching as a supportive covariate-balancing analysis, and (5) sensitivity analysis for unmeasured confounding using the E-value. Baseline characteristics were compared by early seizure status. Continuous variables were summarized as mean ± standard deviation (SD) or median [interquartile range (IQR)] and compared using the t-test or Mann–Whitney U test, as appropriate. Categorical variables were summarized as counts (percentages) and compared using the χ² test or Fisher’s exact test. Unadjusted odds ratios (ORs) for in-hospital mortality were estimated using logistic regression.

**Recovery-dynamics analyses** (primary descriptive objective): To compare early physiological recovery patterns between groups, we analyzed Day 1 (admission) and Day 2 laboratory values using linear mixed-effects models. We focused on markers reflecting metabolic, circulatory, electrolyte, and fluid dynamics: BE, lactate, glucose, serum sodium, and BUN. For each marker, we fit a model with a random intercept for patient ID and fixed effects for early seizure status, time (Day 1 vs. Day 2), and their interaction (seizure × time). The primary inferential target was the group-by-time interaction, indicating differential short-interval trajectories between groups. Estimated marginal means and 95% CIs were derived. These recovery-dynamics analyses were prespecified as exploratory and hypothesis-generating, and we did not apply a formal adjustment for multiple comparisons across biomarkers.

**Mortality analyses** (secondary observational objective): We then constructed prespecified multivariable logistic regression models to explore whether early seizures were associated with in-hospital mortality after adjustment for measured covariates, while recognizing that seizure ascertainment and treatment processes are incompletely captured in the registry. Covariates selected a priori were age, sex, BMI, systolic blood pressure, admission body temperature, and lactate—factors known to be associated with heatstroke severity and prognosis [23–27]. Because lactate could plausibly function as either a confounder or a mediator in this setting, we prespecified two models to provide estimates under different plausible causal assumptions: Model 1 included lactate, and Model 2 excluded lactate. Adjusted ORs and 95% CIs were reported.

We additionally conducted age-stratified multivariable analyses using the same covariate structures, stratifying patients into < 65, 65–74, and ≥ 75 years, to assess the consistency of adjusted associations across age groups (reported in Supplementary Table S1).

Propensity score matching (PSM) was used to improve comparability on measured baseline covariates and to assess whether the seizure–mortality association was directionally consistent under improved covariate balance, rather than to support causal inference.

Our primary propensity score model was prespecified and intentionally parsimonious, prioritizing pre-exposure or early-window variables plausibly related to both the likelihood of early generalized seizures and prognosis, with the goal of confounder-oriented adjustment rather than maximizing prediction of mortality.

To perform matching, we used 1:1 nearest-neighbor propensity score matching without replacement with a caliper width of 0.2 standard deviations of the logit of the propensity score. Propensity scores were estimated via logistic regression with early seizures as the dependent variable and age, sex, BMI, systolic blood pressure, body temperature, lactate, onset location, and time from onset to hospital arrival as predictors. Covariate balance was assessed using standardized mean differences (SMDs), with SMD < 0.1 indicating adequate balance. In the matched cohort, we estimated the association between early seizures and in-hospital mortality using logistic regression with early seizures as the sole predictor.

We did not include GCS in the propensity score model because it is tightly linked to early neurologic presentation and may introduce overadjustment or collider bias in this setting.

We also did not include acute laboratory variables such as pH, base excess, sodium, BUN, or creatinine in the propensity score model because these early derangements are temporally intertwined with seizures and may lie on the causal pathway and/or be influenced by early care processes; instead, we evaluated their short-interval trajectories in the recovery-dynamics analyses.

To address the possibility that baseline health status might contribute to the observed association, we conducted a sensitivity propensity score matching analysis that additionally incorporated available comorbidity indicators (cardiovascular disease, respiratory disease, diabetes mellitus, and psychiatric disease) into the propensity score model; these results are reported in Supplementary Table S2.

Given the possibility of residual confounding in observational research, we calculated the E-value for the primary adjusted estimate to quantify the minimum strength of association that an unmeasured confounder would need to have with both early seizures and in-hospital mortality to fully explain away the observed association [28–30]. E-values were used as sensitivity gauges and were not intended to establish causality.

All statistical analyses were performed using R (version 4.5.2). Linear mixed-effects models were fitted using the lme4 and lmerTest packages, and estimated marginal means were obtained using the emmeans package [30]. Statistical significance was defined as a two-sided p value < 0.05.

## Results

### Baseline characteristics

A total of 3,859 patients (3,451 without early seizures and 408 with early seizures) were included in the descriptive and primary analyses. Early seizures occurred in 10.6% of patients. As shown in Table 1, patients with early seizures were younger, more often male, and more frequently had outdoor onset than those without seizures. On admission, patients with early seizures had higher body temperature and lactate levels. They also exhibited differences in heart rate, respiratory rate, and GCS distributions, consistent with higher physiological stress. In contrast, several laboratory values, including BUN and CRP, were higher in the no–early seizure group (Table 1).

Notably, the substantial age difference between groups in the unmatched cohort underscores the potential for age-related physiologic reserve to confound crude comparisons, motivating the age-adjusted and age-stratified analyses presented below.

**Table 1:** Baseline characteristics of patients with and without early seizures

Variable	No early seizure (*n* = 3451)	Early seizure (*n* = 408)	*p*-value
**Demographics and clinical context**			
Age, years	71.11 ± 17.56	62.84 ± 19.06	< 0.001
Male, %	2343 (67.9)	312 (76.5)	< 0.001
BMI	22.36 ± 4.61	23.39 ± 4.91	< 0.001
Outdoor onset, %	1689 (49.5)	255 (63.4)	< 0.001
Time from onset to hospital, min	285.19 ± 954.63	134.32 ± 316.31	0.002
**On-admission vital signs**			
Systolic BP, mmHg	125.61 ± 22.66	119.75 ± 23.11	< 0.001
Diastolic BP, mmHg	75.69 ± 20.38	72.98 ± 25.29	0.015
Heart rate, bpm	105.96 ± 28.34	124.33 ± 31.92	< 0.001
Respiratory rate, /min	24.92 ± 10.35	27.28 ± 9.26	< 0.001
Body temperature, °C	38.32 ± 1.75	39.19 ± 1.88	< 0.001
GCS score	14 [10–15]	8 [4–14]	< 0.001
**Laboratory findings on admission**			
Lactate, mmol/L	2.66 [1.70–4.40]	3.84 [2.40–6.53]	< 0.001
pH	7.42 ± 0.13	7.40 ± 0.13	0.043
Base excess, mmol/L	−2.11 ± 5.79	−4.47 ± 5.27	< 0.001
Sodium, mEq/L	140.19 ± 7.57	138.15 ± 7.61	< 0.001
Chloride, mEq/L	103.47 ± 7.83	101.54 ± 8.42	< 0.001
Potassium, mEq/L	4.14 ± 0.84	4.22 ± 0.77	0.075
BUN, mg/dL	24.50 [18–37]	21.20 [16–29]	< 0.001
Creatinine, mg/dL	1.40 [0.98–2.10]	1.51 [1.07–2.19]	0.035
WBC (/µL)	10784.99 ± 13060.69	10505.72 ± 9152.08	0.675
Hemoglobin, g/dL	13.63 ± 2.62	13.84 ± 2.44	0.113
Hematocrit, %	40.24 ± 7.39	40.62 ± 6.81	0.326
Platelets (×10^4^/µL)	31.93 ± 50.09	34.56 ± 53.03	0.324
AST, U/L	35 [24–60]	39.50 [26–68.75]	0.001
ALT, U/L	23 [15–40]	26 [17–46]	0.002
Total bilirubin, mg/dL	0.93 [0.70–1.40]	0.90 [0.60–1.33]	0.044
Creatine kinase, U/L	251 [115.50–765.00]	263 [139.00–637.75]	0.456
CRP, mg/dL	0.24 [0.08–1.17]	0.15 [0.06–0.60]	< 0.001
PT activity, %	87 [71–101]	87 [72.50–100]	0.984

Baseline demographic characteristics, clinical context, vital signs, and laboratory findings at admission are summarized according to the presence of early seizures. Continuous variables are presented as mean ± standard deviation (SD) or median [interquartile range, IQR], and categorical variables as number (%). Outcome variables are not included in this table and are reported separately in the Results section

SD, standard deviation; IQR, interquartile range; BMI, body mass index; BP, blood pressure; GCS, Glasgow Coma Scale; BUN, blood urea nitrogen; WBC, white blood cell count; AST, aspartate aminotransferase; ALT, alanine aminotransferase; CK, creatine kinase; CRP, C-reactive protein; PT, prothrombin time

### Day 1–Day 2 recovery dynamics

**Fig. 2:**
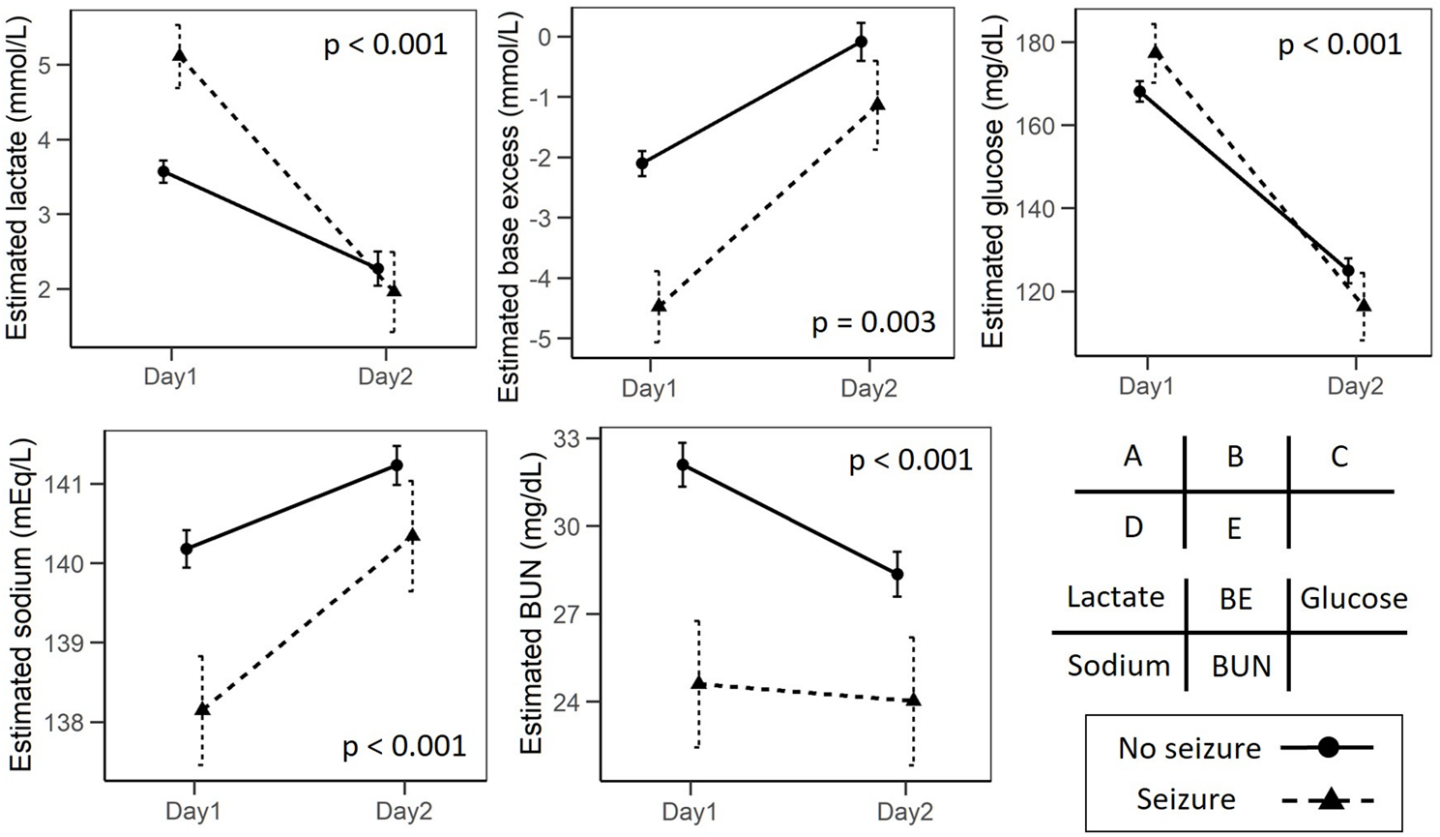
Early recovery dynamics of key metabolic and electrolyte markers according to the presence of early seizures. Estimated marginal means (± 95% confidence intervals) of (A) lactate, (B) base excess, (C) blood glucose, (D) serum sodium, and (E) blood urea nitrogen from Day 1 to Day 2 are shown for patients with and without early seizures. Values were analyzed using linear mixed-effects models with group, time, and group-by-time interaction as fixed effects and a random intercept for each patient. Significant group-by-time interactions indicate differences in the rate of early physiological recovery between groups. BE, base excess; BUN, blood urea nitrogen

Using linear mixed-effects models, we analyzed temporal changes in laboratory parameters from hospital Day 1 (admission) to Day 2. Significant group-by-time interactions between early seizure status and time were observed for five primary markers: BE, lactate, blood glucose, serum sodium, and BUN (Fig. 2).

For lactate, the group-by-time interaction was significant (*p* < 0.001); although patients with early seizures exhibited higher levels on Day 1, the magnitude of decline by Day 2 was greater than that observed in patients without early seizures (Fig. 2A). Similarly, BE showed a significant interaction effect (*p* = 0.003), with the early seizure group presenting with lower BE on Day 1 and demonstrating a larger increase by Day 2 compared with the no–early seizure group (Fig. 2B).

Blood glucose also demonstrated a significant group-by-time interaction (*p* < 0.001): patients with early seizures tended to have higher glucose levels on Day 1, followed by a greater reduction by Day 2 compared with the no–early seizure group (Fig. 2C).

Serum sodium likewise showed a significant group-by-time interaction (*p* < 0.001). Patients with early seizures had lower sodium levels on Day 1, but the increase toward Day 2 was greater than that observed in patients without early seizures (Fig. 2D).

In addition, BUN demonstrated a significant group-by-time interaction (*p* < 0.001). While the no–early seizure group exhibited higher BUN levels on Day 1 with a clear decline by Day 2, the early seizure group had lower Day 1 values and a smaller subsequent decrease (Fig. 2E), indicating distinct short-interval trajectories between groups.

In addition to these primary markers, temporal changes in body temperature, creatine kinase, and platelet count from Day 1 to Day 2 were also evaluated using linear mixed-effects models (Supplementary Figure S1) as exploratory analyses. Significant group-by-time interactions were observed for body temperature, as well as for CK and platelet count. By contrast, no significant group-by-time interactions were observed for pH, PT activity, creatinine, WBC, CRP, hematocrit, or total bilirubin, all of which showed broadly similar temporal trends in both groups. Collectively, these Day 1–Day 2 trajectories delineate a distinct short-interval recovery pattern characterized by greater early perturbation with subsequent normalization in several metabolic and electrolyte markers among patients with early seizures.

### Logistic regression analyses

Following the characterization of short-interval recovery patterns, we evaluated the association between early seizures and in-hospital mortality. We specifically considered that in the most moribund patients, early seizures might be less likely to be observed due to profound systemic exhaustion or early mortality; thus, crude comparisons could be masked by negative confounding.

In the unmatched cohort, in-hospital mortality was 180/3,451 (5.2%) among patients without early seizures and 27/408 (6.6%) among those with early seizures. ICU length of stay was longer in patients with early seizures, and crude in-hospital mortality was numerically higher; however, neither difference reached statistical significance. In unadjusted logistic regression, early seizures were not significantly associated with in-hospital mortality (OR 1.29, 95% CI 0.85–1.96) (Table 2).

In prespecified multivariable models, early seizures showed an inverse association with in-hospital mortality after adjustment for measured covariates (Model 1 [lactate included]: aOR 0.48, 95% CI 0.27–0.86; Model 2 [lactate excluded]: aOR 0.57, 95% CI 0.33–0.99). Established severity-related covariates retained expected associations with mortality across models. These adjusted estimates should be interpreted as marker–outcome associations in an observational registry; differential ascertainment of early seizures in the most severely ill patients cannot be excluded.

Given the younger age distribution in patients with early seizures in the unmatched cohort (Table 1), we additionally performed age-stratified multivariable analyses using the same prespecified covariate structures as the primary models (< 65, 65–74, and ≥ 75 years).

Across strata, the adjusted odds ratios remained below 1 in both models, but confidence intervals were wide and statistical significance was not reached within individual strata; detailed estimates are provided in Supplementary Table S1.

**Table 2:** Association between early seizures and in-hospital mortality

Variable	Odds ratio	95% CI (lower)	95% CI (upper)	*p*-value
**Crude model**				
Early seizure	1.29	0.85	1.96	0.236
Age (per 1-year increase)	1.01	1.00	1.02	0.007
Male	1.01	0.75	1.37	0.928
BMI	1.04	1.01	1.07	0.012
Body temperature (°C)	1.68	1.54	1.84	< 0.001
Systolic BP (per 10 mmHg)	0.814	0.785	0.844	< 0.001
Lactate (mmol/L)	1.15	1.11	1.18	< 0.001
**Model 1 (lactate included)**				
Early seizure	0.483	0.273	0.855	0.012
Age (per 1-year increase)	1.02	1.01	1.04	< 0.001
Male	1.32	0.883	1.98	0.175
BMI	1.06	1.03	1.10	< 0.001
Body temperature (°C)	1.56	1.40	1.74	< 0.001
Systolic BP (per 10 mmHg)	0.860	0.820	0.902	< 0.001
Lactate (mmol/L)	1.12	1.07	1.16	< 0.001
**Model 2 (lactate excluded)**				
Early seizure	0.571	0.331	0.986	0.044
Age (per 1-year increase)	1.02	1.01	1.03	0.003
Male	1.34	0.909	1.96	0.141
BMI	1.05	1.02	1.09	0.003
Body temperature (°C)	1.57	1.42	1.75	< 0.001
Systolic BP (per 10 mmHg)	0.838	0.802	0.877	< 0.001

Crude and adjusted odds ratios (ORs) with 95% confidence intervals (CIs) for in-hospital mortality associated with early seizures are shown. Multivariable logistic regression models were adjusted for age, sex, body mass index, systolic blood pressure, body temperature, and lactate level (Model 1) or without lactate (Model 2)

OR, odds ratio; CI, confidence interval; BMI, body mass index; BP, blood pressure

### Propensity score matching

Propensity score matching yielded 287 well-balanced pairs. Covariate balance improved substantially across baseline variables, with most SMDs reduced to below conventional thresholds (Table 3). In the matched cohort, early seizures were associated with lower in-hospital mortality (OR 0.37, 95% CI 0.18–0.76).

In a sensitivity matching analysis that additionally incorporated available comorbidity indicators (cardiovascular disease, respiratory disease, diabetes mellitus, and psychiatric disease) into the propensity score model, 286 pairs were formed (Supplementary Table S2).

In this sensitivity-matched cohort, in-hospital mortality was 29/286 (10.1%) in the no–early seizure group and 12/286 (4.2%) in the early seizure group (*p* = 0.009). The directional consistency of these results across both primary and sensitivity matching strategies reinforces the hypothesis that early seizures identify a clinical phenotype with distinct recovery potential, independent of measured baseline severity, comorbidities, or the time from onset to hospital arrival.

**Table 3:** Covariate balance before and after propensity score matching

Variable	No early seizure (*n* = 287)	Early seizure (*n* = 287)	*p*-value	SMD
**Demographics and clinical context**				
Age, years	64.78 ± 18.34	63.75 ± 18.67	0.505	0.056
Male, %	228 (79.4)	219 (76.3)	0.421	0.076
Outdoor onset, %	195 (67.9)	189 (65.9)	0.657	0.044
BMI	23.11 ± 4.87	23.20 ± 4.94	0.830	0.018
Time from onset to hospital, min	51.00 [36.50–86.00]	54.00 [37.00–90.50]	0.619	0.054
**On-admission vital signs**				
Systolic BP, mmHg	118.09 ± 31.82	120.33 ± 34.58	0.421	0.067
Diastolic BP, mmHg	70.90 ± 21.12	72.98 ± 25.98	0.294	0.088
Heart rate, bpm	119.63 ± 30.68	124.97 ± 31.12	0.040	0.173
Respiratory rate, /min	27.06 ± 8.79	26.90 ± 8.93	0.832	0.018
Body temperature, °C	39.17 ± 1.80	39.24 ± 1.85	0.657	0.037
GCS score	13 [6–14]	7 [4–14]	< 0.001	0.356
**Laboratory findings on admission**				
Lactate, mmol/L	3.90 [2.23–6.55]	3.88 [2.44–6.45]	0.950	0.055
pH	7.41 ± 0.11	7.41 ± 0.11	0.810	0.020
Base excess, mmol/L	−3.43 ± 9.45	−4.11 ± 4.70	0.280	0.091
HCO₃⁻, mmol/L	20.60 [17.10–24.00]	20.30 [16.80–22.90]	0.194	0.107
Sodium, mEq/L	138.92 ± 7.62	138.15 ± 7.74	0.232	0.100
Chloride, mEq/L	102.24 ± 7.55	101.63 ± 8.50	0.368	0.076
Potassium, mEq/L	4.32 ± 1.06	4.19 ± 0.78	0.115	0.132
BUN, mg/dL	21.90 [16–30.08]	21.20 [16.50–28.65]	0.481	0.170
Creatinine, mg/dL	1.50 [1.12–2.10]	1.52 [1.04–2.20]	0.713	0.077
WBC (/µL)	12,168 ± 19,002	10,258 ± 8806	0.123	0.129
Hemoglobin, g/dL	13.95 ± 2.60	13.60 ± 2.38	0.100	0.138
Hematocrit, %	41.00 ± 7.44	39.92 ± 6.51	0.067	0.154
Platelets (×10^4^/µL)	21.90 [16.15–28.50]	21.10 [16.58–27.82]	0.616	0.090
AST, U/L	39 [26–68]	40 [26–66.75]	0.909	0.152
ALT, U/L	26 [15–47]	27 [17–43.75]	0.990	0.109
Total bilirubin, mg/dL	0.90 [0.67–1.40]	0.90 [0.60–1.30]	0.158	0.075
Creatine kinase, U/L	265 [123–775]	247.5 [132–645]	0.406	0.139
CRP, mg/dL	0.18 [0.06–0.70]	0.14 [0.05–0.60]	0.382	0.036
PT activity, %	87.90 [71–100]	87.00 [73–100]	0.840	0.078

Standardized mean differences (SMDs) for baseline covariates before and after 1:1 nearest-neighbor propensity score matching are presented. Adequate balance was defined as an SMD less than 0.1 for each covariate. Continuous variables are presented as mean ± standard deviation (SD) or median [interquartile range, IQR]

SMD, standardized mean difference; SD, standard deviation; IQR, interquartile range; BMI, body mass index; BP, blood pressure; GCS, Glasgow Coma Scale; BUN, blood urea nitrogen; WBC, white blood cell count; AST, aspartate aminotransferase; ALT, alanine aminotransferase; CK, creatine kinase; CRP, C-reactive protein

### Sensitivity analysis for unmeasured confounding

To assess the robustness of the observed association between early seizures and survival to potential unmeasured confounding, we performed a sensitivity analysis using the E-value. In the multivariable model including lactate (Model 1), early seizures were associated with lower in-hospital mortality (adjusted odds ratio [aOR] 0.48, 95% confidence interval [CI] 0.27–0.86). The corresponding E-value for this adjusted estimate was 2.36, indicating that an unmeasured confounder would need to be associated with both early seizures and in-hospital mortality by a risk ratio of at least 2.36 to fully explain away the observed association. To shift the upper bound of the 95% confidence interval (aOR 0.86) to the null, an unmeasured confounder with a risk ratio of approximately 1.47 would be required.

The directional consistency of the association was also maintained in the model excluding lactate (Model 2: aOR 0.57, 95% CI 0.33–0.99), with a corresponding E-value of 2.04. The E-value for the upper limit of the 95% confidence interval was 1.18. These findings suggest that the observed association between early seizures and survival, while strictly hypothesis-generating, remains moderately robust against potential residual confounding, even when accounting for the possible masking effects of systemic metabolic distress.

## Discussion

Using a large nationwide multicenter registry, we primarily characterized early short-interval physiologic trajectories in heat-related illness according to clinically documented early generalized seizures. As a secondary aim, we descriptively evaluated associations with clinical outcomes, including in-hospital mortality. Across several metabolic and electrolyte markers, patients with early seizures demonstrated a pattern of greater initial perturbation on Day 1 accompanied by more rapid short-interval normalization by Day 2. This pattern may not be fully explained by baseline severity alone and may reflect heterogeneity in early short-interval physiologic trajectories associated with early seizures [31, 32]. Importantly, the overall clinical implication is that clinically documented early heat-related seizures should not be automatically or uniformly interpreted at initial presentation as indicating a more severe condition or a worse short-term outcome. Given the registry’s limited temporal resolution and the absence of detailed neurologic phenotyping or treatment-timing data, these findings should be interpreted as descriptive, hypothesis-generating evidence of heterogeneity in early recovery dynamics rather than as mechanistic proof.

Notably, the short-interval differences were detected mainly in metabolic and fluid/electrolyte–related markers, whereas inflammatory and coagulation markers showed broadly similar early trends. A plausible explanation is that metabolic and electrolyte abnormalities can shift over hours with changes in perfusion, cooling, and fluid/electrolyte management, making between-group differences more detectable within a Day 1–Day 2 window. In contrast, commonly used clinical markers such as CRP and PT activity may reflect downstream consequences of upstream cytokine responses and endothelial injury and can therefore exhibit a temporal lag relative to early metabolic changes [33]. In addition, these markers have variable kinetics and are sensitive to sampling-time variability and early care processes, so divergence may be difficult to capture using only two early discrete time points. These considerations do not exclude the possibility of later divergence in inflammatory or coagulation pathways; rather, they underscore the need for studies with longer follow-up and finer-grained temporal profiling.

The observed trajectories of serum sodium and BUN are also consistent with potentially reversible disturbances in fluid, electrolyte, and osmotic balance being temporally linked to clinically documented early seizures [34]. Patients with early seizures had lower sodium levels on Day 1 and exhibited larger increases by Day 2, suggesting that acute hyponatremia may have lowered the seizure threshold in a subset of patients [35–37]. In heat-related illness, fluid intake behaviors before presentation and early resuscitative strategies can substantially influence electrolyte homeostasis [38]. Accordingly, early seizures may accompany acute osmotic or metabolic disturbances and should not be assumed to uniformly indicate irreversible neurologic injury. However, these observations do not permit mechanistic inference, given the absence of neurologic phenotyping and treatment-level data that would be required to support such interpretation (e.g., EEG confirmation; seizure burden and timing; sedation depth; and the timing and type of antiseizure medications and other early interventions). Therefore, the sodium and BUN patterns should be interpreted as short-interval, hypothesis-generating phenotypic features temporally associated with early seizures, not as evidence that seizures themselves drive recovery or that the observed changes can be attributed to any specific intervention.

Having established the primary descriptive finding of differential Day 1–Day 2 trajectories, we then performed a secondary, observational analysis of in-hospital mortality. This analysis was intended to contextualize the trajectory patterns rather than to support practice-informing or causal conclusions. Although crude comparisons did not show a significant association, adjusted analyses (prespecified multivariable models and supportive covariate-balancing analyses using propensity score matching) yielded an inverse association between early seizures and mortality. Importantly, this pattern is biologically more plausibly explained by residual and negative confounding—such as differential ascertainment of seizures, closer monitoring or treatment intensity, and survivorship-related selection—than by any protective effect of seizures themselves.　Accordingly, our mortality findings should be read as marker–outcome associations that help frame the above trajectory differences, not as evidence that early seizures causally improve survival.

We also considered whether the observed inverse association might be attributable to earlier discovery and transport among patients who developed seizures. However, time from symptom onset to hospital arrival was explicitly incorporated into the propensity score model, and the association between early seizures and lower mortality persisted after matching. This suggests that earlier transport alone is unlikely to fully account for the observed relationship, while alternative explanations remain plausible in observational data. In particular, differential monitoring intensity, sedation practices, treatment timing, and other unmeasured aspects of early care processes may contribute to the observed associations.

To further assess the robustness of our findings, we evaluated the potential impact of unmeasured confounding using E-values. For the model excluding lactate (adjusted odds ratio 0.57), the E-value was 2.04, indicating that an unmeasured confounder would need to be associated with both early seizures and in-hospital mortality by a risk ratio of approximately 2.04 each to fully explain away the observed adjusted association. The E-value for the upper limit of the 95% confidence interval was 1.18, suggesting that this specification may be more sensitive to modest unmeasured confounding. Therefore, the E-value is best read as a sensitivity gauge that quantifies how strong unmeasured confounding would need to be under a given model specification, rather than as evidence that the association is causal or that mechanism can be attributed to early seizures.

Taken together, our findings support the interpretation that clinically documented early generalized seizures in heat-related illness should be understood as an observational marker associated with outcomes and early short-interval trajectories, rather than as a uniform indicator of irreversible neurologic injury or as evidence of a protective effect. These results may help calibrate early risk assessment at initial presentation and avoid premature prognostic pessimism. At the same time, the findings remain hypothesis-generating and warrant prospective validation with higher-resolution neurologic phenotyping (including seizure timing and burden and EEG), more granular measurement of early care processes (including sedation and treatment timing), and longer-term follow-up to clarify mechanisms, care-process interactions, and clinical implications.

### Limitations

This study has several important limitations. First, as a retrospective observational study, causal inference cannot be established. Although we employed multivariable adjustment, propensity score matching, and sensitivity analyses to mitigate confounding, residual confounding cannot be entirely excluded. In particular, any inverse association between early seizures and mortality is confounding-vulnerable and may reflect residual and negative confounding (including survivorship-related bias), rather than any protective effect of seizures. Because seizure occurrence and documentation depend on observation opportunity, the most profoundly ill patients may have been less likely to manifest or have seizures recorded due to global physiologic exhaustion, deep sedation, or early death prior to assessment, which could bias crude and adjusted estimates.

Second, owing to inherent limitations of the registry-based design, early seizures were ascertained from routine prehospital and emergency department documentation without electroencephalographic confirmation; therefore, exposure misclassification cannot be excluded. Specifically, data on seizure subtype, duration, presence of nonconvulsive seizures, and the timing and type of antiseizure medications were unavailable. In addition, the registry does not include standardized information on seizures occurring after the initial emergency department assessment; therefore, we could not evaluate late-onset seizures separately. Acute and intensive care interventions were performed at the discretion of each participating institution. These interventions included cooling methods, fluid resuscitation strategies, sedation and anticonvulsant use, and ventilatory management. As a result, we could not systematically assess heterogeneity in treatment intensity or timing, and such unmeasured variation may have influenced neurological outcomes and the observed recovery dynamics. Moreover, the registry does not capture key neurologic and care-process granularity—such as seizure burden, timing relative to physiologic deterioration, EEG monitoring practices, sedation depth, or withdrawal-of-care decisions—which substantially limits mechanistic interpretation and may contribute to confounding.

Third, the registry does not provide standardized information on hyperthermia exposure duration or time above specific temperature thresholds; therefore, residual confounding related to time-dependent heat exposure cannot be excluded.

Fourth, our analyses focused on early physiological changes through hospital Day 2 and did not evaluate subsequent inflammatory progression, persistent organ dysfunction, or long-term neurological and functional outcomes. Accordingly, the reported Day 1–Day 2 trajectories should be interpreted as short-interval descriptive patterns rather than evidence of causal recovery mechanisms. Although an inverse association with short-term mortality was observed after adjustment in some model specifications, the longer-term prognostic implications and their relationship to unmeasured care processes remain to be clarified.

Finally, this analysis was based on a multicenter Japanese registry, and the findings may not be directly generalizable to healthcare systems with different epidemiological profiles and treatment pathways for heat-related illness.

## Conclusion

Clinically documented early generalized seizures in heat-related illness were associated with a distinct short-interval trajectory of metabolic and electrolyte markers over the first two hospital days. In secondary, observational analyses, an inverse association with in-hospital mortality was observed after multivariable adjustment and covariate-balancing approaches; however, this finding should be interpreted conservatively and is confounding-vulnerable, including to differential seizure ascertainment. Overall, the primary clinical implication is that early heat-related seizures should not be automatically regarded as uniformly ominous at initial presentation. These results are hypothesis-generating and warrant prospective validation with higher-granularity neurologic phenotyping and treatment-timing data.

## Supplementary Information

Supplementary Material 1

Supplementary Material 2

Supplementary Material 3

## Data Availability

The datasets used and/or analyzed during the current study available from the corresponding author on reasonable request.

## Abbreviations

aOR: Adjusted odds ratio
AST: Aspartate aminotransferase
ALT: Alanine aminotransferase
BE: Base excess
BMI: Body mass index
BP: Blood pressure
BUN: Blood urea nitrogen
CI: Confidence interval
CK: Creatine kinase
CRP: C-reactive protein
EEG: Electroencephalogram
E-value: E-value for sensitivity analysis of unmeasured confounding
GCS: Glasgow Coma Scale
JAAM-HsS: Japanese Association for Acute Medicine Heatstroke and Hypothermia Surveillance
ICU: Intensive care unit
IQR: Interquartile range
OR: Odds ratio
PSM: Propensity score matching
PT: Prothrombin time
SD: Standard deviation
SMD: Standardized mean difference
WBC: White blood cell
