# Supplementary material for: Early seizures and heterogeneity of physiologic recovery in heat-related illness: a nationwide registry study
Source: Crit Care. 2026 Mar 20;30:220. doi: 10.1186/s13054-026-05961-7 (PMC13126818; doi:10.1186/s13054-026-05961-7)
Supplement: Supplementary file 1 — Supplementary Material 1 [file 13054_2026_5961_MOESM1_ESM.pdf]

Figure S1. Early changes in laboratory and physiological parameters according to the presence of early seizures

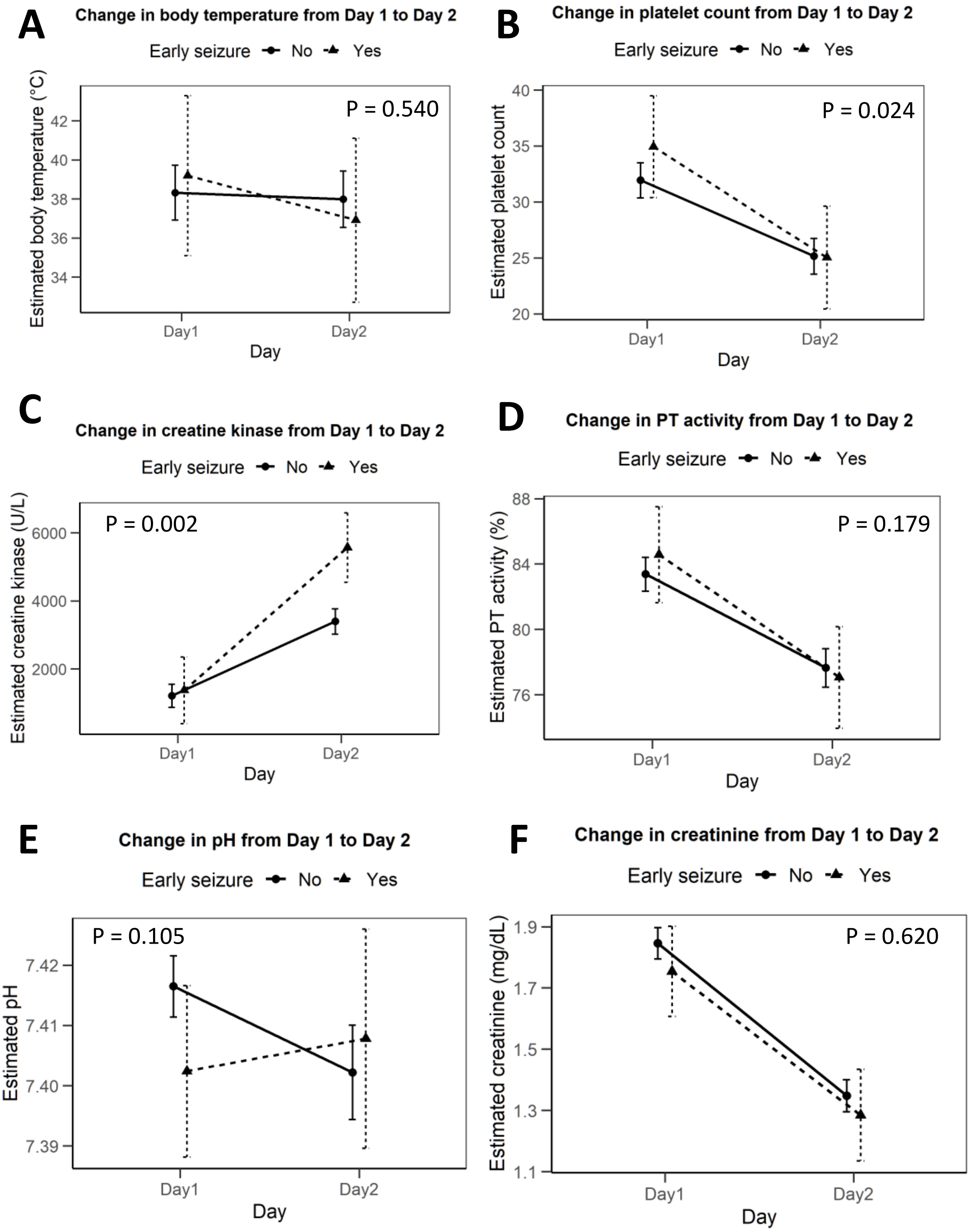

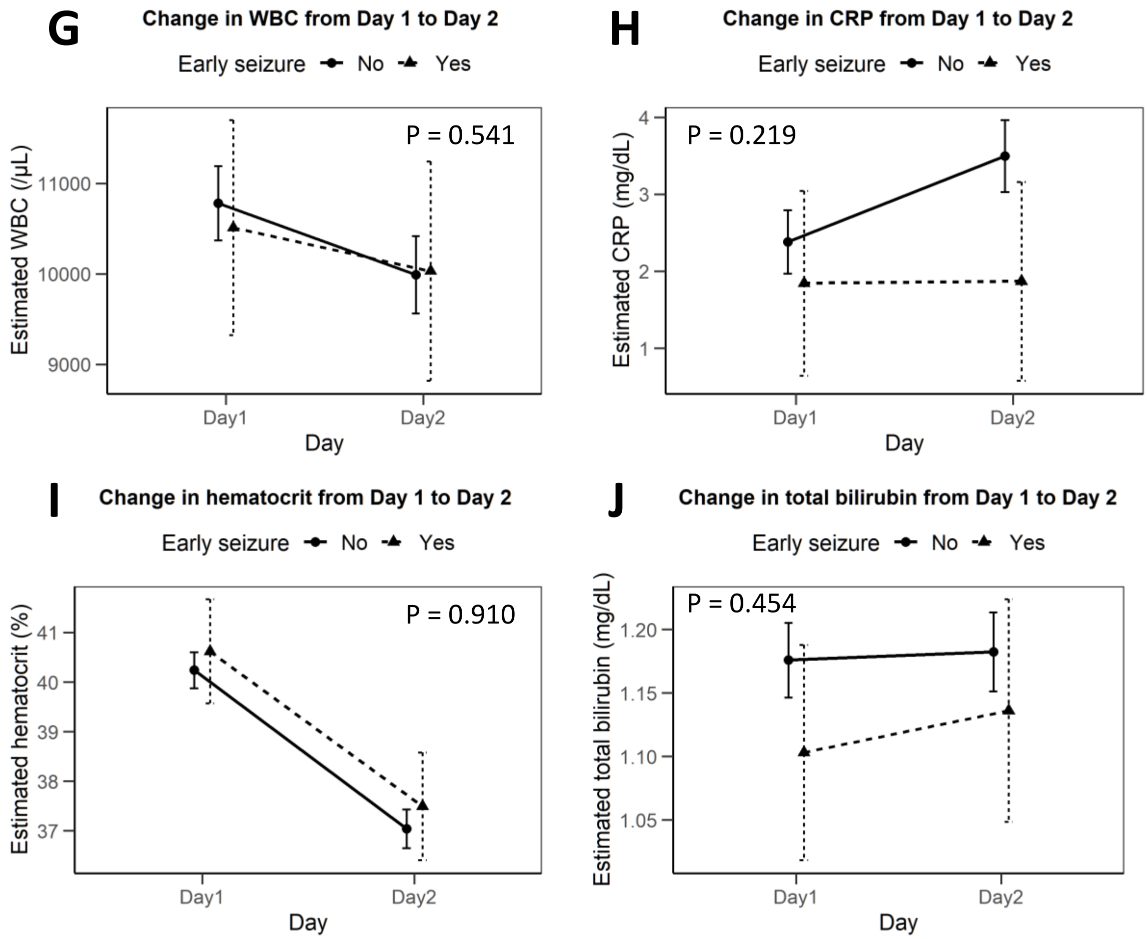

Estimated marginal means of laboratory and physiological parameters on Day 1 and Day 2 are shown for patients with and without early seizures. Panels display changes in (A) body temperature, (B) platelet count, (C) creatine kinase, (D) PT activity, (E) pH, (F) creatinine, (G) white blood cell count (WBC), (H) C-reactive protein (CRP), (I) hematocrit, and (J) total bilirubin. Values are presented as estimated marginal means with 95% confidence intervals derived from linear mixed-effects models. Lines represent group-specific trajectories from Day 1 to Day 2. The p-values displayed within each panel indicate the statistical significance of the group-by-time interaction term (early seizure status × Day), testing whether the change from Day 1 to Day 2 differs between patients with and without early seizures. Figure S1 Abbreviations: PT, prothrombin time; WBC, white blood cell count; CRP, C-reactive protein.
